# How many sites are enough? a novel, site-based power analysis method for real-world registry studies of anti-amyloid monoclonal antibodies

**DOI:** 10.1016/j.jarlif.2025.100020

**Published:** 2025-07-22

**Authors:** Kenichiro Sato, Yoshiki Niimi, Ryoko Ihara, Atsushi Iwata, Takeshi Iwatsubo

**Affiliations:** a Department of Neuropathology, Graduate School of Medicine, The University of Tokyo, Hongo 7-3-1, Bunkyo-ku, Tokyo 113-8655, Japan; b Unit for Early and Exploratory Clinical Development, The University of Tokyo Hospital, Hongo 7-3-1, Bunkyo-ku, Tokyo 113-8655, Japan; c Dementia Inclusion and Therapeutics, The University of Tokyo Hospital, Hongo 7-3-1, Bunkyo-ku, Tokyo 113-8655, Japan; d Department of Neurology, Tokyo Metropolitan Institute for Geriatrics and Gerontology, Sakaecho 35-2, Itabashi-ku, Tokyo 173-0015, Japan

**Keywords:** Real-world registries, Power analysis, Anti-amyloid monoclonal antibodies, Amyloid-related imaging abnormalities

## Abstract

**Background:**

Real-world registries ALZ-NET (US) and AD-DMT (Japan) support safety surveillance of anti-amyloid antibodies. Conventional power calculations—dividing required patients by mean per-site caseload—can underestimate the number of centers needed because of patient counts variability.

**Objectives:**

To develop and evaluate a simulation-based method for site-level sample size planning that incorporates inter-site variability.

**Design:**

We developed a simulation using a zero-truncated negative binomial model to reflect caseload heterogeneity. We estimated the required sites (k) to achieve a target precision (95 % confidence interval [CI] width) for ARIA incidence under random and volume-weighted sampling, based on data from published trials. The required number of sites was determined as the point where the CI width met a prespecified precision target (< 0.1).

**Setting:**

Simulated ALZ-NET and AD-DMT registry settings using prevalence and ARIA frequencies from published lecanemab and donanemab trials.

**Measurements:**

Precision (95 % CI width) for estimating ARIA incidence in *APOE*-ε4 homozygotes; comparison of required site counts as estimated by the three methods.

**Results:**

Under random sampling, our method’s site requirement (∼320 sites) was consistent with the ICC-adjusted method, whereas the conventional method underestimated the need (∼220 sites). Critically, our framework showed that strategic volume-weighted sampling could reduce the requirement to as few as 110 sites, surpassing the efficiency of the static analytical methods.

**Conclusions:**

Conventional methods risk underestimating site requirements by ignoring caseload heterogeneity. Our simulation framework provides more realistic estimates and, crucially, quantifies the substantial efficiency gains from strategic recruitment, serving as a flexible tool to optimize registry design.

## 1 Introduction

The recent regulatory approval of anti-amyloid monoclonal antibodies such as lecanemab [1] and donanemab [2] has created an urgent need for robust, real-world safety monitoring of Amyloid-Related Imaging Abnormalities (ARIA), which occur more frequently in *APOE*-ε4 homozygotes [3]. While pivotal clinical trials reported genotype-specific ARIA-E and ARIA-H rates, these estimates were derived from selected centers under protocolized conditions.

As of February 2025, approximately 13,500 American patients have been treated with lecanemab in 1200 facilities in the United States and approximately 6800 Japanese patients have been treated with lecanemab in 660 facilities in Japan [4]. Nationwide real-world registries (e.g., ALZ-NET in the United States (https://www.alz-net.org) [5] and AD-DMT Clinical Registry in Japan (https://addmt.ncnp.go.jp)) have been established to capture these patients. Since many patients are recruited to these registries through invitations from participating sites, maximizing site participation is crucial to enroll a sufficient number of patients for robust safety analyses.

The total number of patients needed to achieve a research objective, such as detecting an effect size with adequate statistical power or estimating a parameter with sufficient precision, is typically determined using sample size calculations. For studies recruiting through participating centers, such as registries related to disease-modifying therapies (DMTs), estimating *the required number of centers* is a key practical consideration. A common approach is to divide the total required number of patients by an estimated average number of patients per center. For example, if the required sample size for a specific analysis is calculated to be 300 patients, and the estimated number of patients per site is roughly 10 on average, then including approximately 30 sites would seem to suffice.

However, patient distribution across centers is typically uneven, influenced by factors like facility size, specialist availability, and capacity, especially for specialized treatments like DMTs. Similar to findings from cluster-randomized trials, where increased variability in facility-level cluster size reportedly leads to reduced power or precision [6,7], this variability in treated-patient counts per center means that simply dividing by an average may underestimate the number of centers needed to achieve the target enrollment and adequate statistical power or precision in real-world settings.

This study proposes a novel, simulation-based method for site-based sample size planning in real-world data (RWD) studies. Assuming patient counts per center (≥ 1) follow a zero-truncated negative binomial (ZTNB) distribution [8] to reflect real-world overdispersion in DMT-treated patient numbers across facilities, we compare our proposed method with two analytical approaches—a conventional average-based calculation and a method adjusted for data clustering using an intra-class correlation coefficient (ICC)—to assess potential discrepancies in estimating the required number of participating centers.

## 2 Methods

### 2.1 Framework for precision-based sample size estimation

The primary outcome was to determine the number of participating required to estimate the incidence of ARIA in APOE-ε4 homozygotes with a desired level of statistical precision. Precision was quantified by the width of the 95 % confidence interval (CI) for the ARIA incidence rate. To this end, we compared three distinct methods for estimating the required number of facilities: (a) proposed simulation-based method, a Monte Carlo simulation approach that directly models the site-to-site heterogeneity in patient caseloads, which is characteristic of real-world registry settings, (b) conventional analytical method, a standard analytical approach that calculates precision based on the total number of patients, but ignores the clustered nature of the data (i.e., patients being nested within facilities), and (c) ICC-adjusted analytical method, a widely used analytical method that adjusts for data clustering using a pre-specified intra-class correlation coefficient (ICC) and the average facility size [9]. For illustrative purposes in our results, we will use a benchmark CI width of 0.10 (corresponding to an estimate with a margin of error of ±5 %) to compare the number of facilities required under different methodologies and scenarios.

### 2.2 Proposed simulation-based method

Our simulation framework was designed to emulate a real-world, multi-center registry. First, we fixed a total patient population, *N_total_*, to be distributed across *n_sites_* candidate facilities. To model the significant variability in patient caseloads observed in clinical practice, we drew the patient count for each facility, *n_i_*, from a zero-truncated negative binomial (ZTNB) distribution, which captures overdispersion effectively. The site caseloads were generated as follows:

First, unnormalized site weights *w_i_* were drawn from a ZTNB distribution:

$$w_{i}∼ZTNB\left(size\in \left\{0.5,1\right\},\;\mu =\left(N_{total}/n_{sites}\right)\times \;\left\{0.5,1\right\}\right).$$

Then each site’s share of *N_total_* was allocated proportionally, with a final adjustment to ensure the total number of patients equals *N_total_*:

$$n_{i}=round\left(\frac{w_{i}}{\sum_{j}w_{j}}N_{total}\right)\text{,}\;\text{such}\;\text{that}\;\sum_{i}n_{i}=N_{total}.$$

Within each simulated facility *i* with *n_i_* patients, we assumed a fraction *a_homo_* of patients were APOE-ε4 homozygotes, among whom the ARIA incidence was set to *p_2_*, derived from published trial data for lecanemab and donanemab [1,2].

The simulation proceeded as follows for a given number of selected facilities, *k*:

1. **Facility Sampling**: From the population of nsites facilities, k distinct facilities were sampled without replacement. To simulate different recruitment strategies, each site was assigned a selection probability proportional to niαpow, where αpow=0 corresponds to uniform random sampling and αpow>0 gives preference to recruiting high-volume sites.

2. **Precision Calculation:** This process was repeated for 1000 Monte Carlo replicates. In each replicate:

• The total number of APOE-ε4 homozygotes (*n_2_*) in the *k* sampled facilities was aggregated.
• The total number of ARIA events among these homozygotes was determined based on the incidence rate *p_2_*.
• The 95 % CI for the incidence rate *p_2_* was calculated using the standard Wald method for a binomial proportion.

3. **Summarizing Precision:** After 1000 replicates, the distribution of the 95 % CI widths was summarized to obtain the median CI width and its 95 % empirical range for each value of *k*. This allows for the determination of the number of facilities needed to achieve a target precision level.

### 2.3 Analytical methods

For comparison, we calculated the precision using two analytical approaches. These methods calculate the required number of patients (*m*) first, which is then converted to an equivalent number of facilities (*k*) by dividing by the average caseload (*k* = *m*/(*N_total_*/*n_sites_*)).

#### 2.3.1 Conventional analytical method

This approach ignores the clustering of patients within facilities and calculates precision assuming a simple random sample (without ICC-adjustment). For a given number of total patients (*m*), the number of APOE-ε4 homozygotes is *n_2_* = *m* × *a_homo_*. The 95 % CI for the ARIA incidence *p_2_* is calculated as *p_2_* ± 1.96 × SE, where the standard error (SE) is: $SE=\sqrt{p_{2}\left(1-p_{2}\right)/n_{2}}$.

The full CI width is therefore 2 × 1.96 × SE. This method serves as a naive baseline, representing the most optimistic scenario.

#### 2.3.2 ICC-Adjusted analytical method

This is the standard approach to account for data clustering. It adjusts the variance from the conventional method using a Design Effect (Deff). The Deff is calculated as:

$$Deff=1+\left(\bar{m}-1\right)\times ICC$$

where $\bar{m}$ is the average number of patients per facility (*N_total _/n_sites _*) and the ICC is a pre-specified value representing the degree of similarity among patients within the same facility. The standard error from the conventional method is inflated by a factor of $\sqrt{Deff}$, leading to a wider CI. We performed sensitivity analyses using a range of plausible ICC values.

### 2.4 Parameter settings

The parameters for the simulations and analytical calculations were based on published data from the lecanemab and donanemab clinical trials (Table S1).

• **Patient and Facility Numbers:** Total patient population (*N_total_*) ranged from 6000 to 30,000, distributed across a total number of facilities (*n_sites_*) ranging from 600 to 1500.
• **Genotype and ARIA Rates:** The prevalence of *APOE*-ε4 homozygotes (*a_homo_*) was varied between 10 %, 15 %, and 20 %. The baseline ARIA incidence rates (*p_2_*) for homozygotes were sourced from trial data (e.g., Lecanemab/ARIA-E: *p_2_* = 32.6 %; Donanemab/ARIA-E: *p_2_* = 40.6 %) and varied by factors of 0.9, 1.0, and 1.1 in sensitivity analyses.
• **Simulation Parameters:** The ZTNB distribution was parameterized by a size parameter (0.5 or 1) and a mean (*μ*) set at 0.5, 1, or 2 times the average caseload *N_total_/n_sites_*. The site selection weighting exponent (*α_pow_*) was set to 0, 1, or 2.
• **ICC Values:** For the ICC-adjusted method, ICC was set to 0.01, 0.025, or 0.05.

### 2.5 Parameter influence analysis

To systematically evaluate the factors influencing the estimation of precision, we fit a linear mixed-effects model. The dependent variable (*Y_ij_*) was the 95 % CI width for the ARIA incidence rate. A conceptual representation of the model is:

Here, for a given set of conditions *i* and number of facilities *k_ij_*, the model estimates the CI width (*Y_ij_*). *β*_0_ is the overall intercept, and *β_1_* is the average slope for the number of facilities *k* (i.e., the average change in precision per additional facility). b_0i_ and b_1i_ are random effects for the intercept and slope, respectively, allowing them to vary across different panels of conditions. The terms *β_p_X_p,i_* represent the fixed effects of other predictors, such as the estimation method used, *N_total_, n_sites_, a_homo_*, and simulation-specific parameters. This model allows for a comprehensive comparison of how different real-world factors and methodological assumptions affect the required number of facilities for robust safety monitoring.

### 2.6 Ethics

This study was approved by the University of Tokyo Graduate School of Medicine Institutional Ethics Committee (ID: 2024264NIe-(1)). No informed consent was required as it uses publicly available data only.

## 3 Results

### 3.1 Precision as a function of facility number and recruitment strategy

**Fig. 1:**
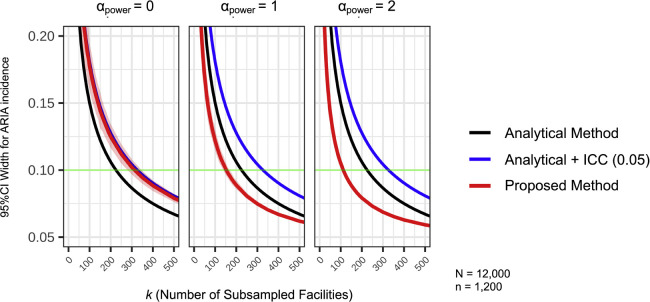
**Comparison of Methods for Estimating Required Facilities for a Target Precision**. A comparison of three methods for estimating the required number of facilities to achieve a target precision under different recruitment strategies. The vertical axis shows the 95 % confidence interval (CI) width for ARIA incidence, where a smaller width indicates higher precision. The horizontal axis is the number of participating facilities (*k*). The curves compare the proposed simulation-based method (red) with two analytical alternatives: a conventional analytical method (black) and an analytical method adjusted for data clustering (intra-class correlation coefficient = 0.05, blue). The panels illustrate the impact of different recruitment strategies: random sampling (α_power_ = 0) versus volume-weighted sampling (α_power_ ​= 1 and α_power_ ​= 2). The shaded red ribbon represents the 95 % empirical range of the simulation-based estimates. The horizontal green line indicates a benchmark precision level (95 % CI width = 0.10). Results are shown for a representative scenario (lecanemab, ARIA-E, with *N* = 12,000, *n* = 1200, *a_2_* = 0.15, *Adjust p_2_* = 1.0, *ZTNB size* = 0.5, and *ZTNB* μ = 0.5 × *N/n*).

Abbreviations: ARIA, Amyloid-Related Imaging Abnormalities; CI, confidence interval.

Our simulations first demonstrated that statistical precision improved—indicated by a smaller 95 % CI width—as the number of participating centers (*k*) increased. We then compared the point estimates of the number of facilities required by the three different methods to achieve a benchmark precision level (95 % CI width = 0.10), as illustrated in a representative scenario (lecanemab, ARIA-E, with *N* = 12,000, *n* = 1200, *a_2_* = 0.15, *Adjust p_2_* = 1.0, *ZTNB size* = 0.5, and *ZTNB* μ = 0.5 × *N/n*) (Fig. 1).

The analytical methods produced static estimates. The conventional analytical method suggested that approximately 220 facilities would be required to reach the target. When this method was adjusted for data clustering using an ICC of 0.05, the analytical + ICC method yielded a higher estimate of approximately 320 facilities.

Our proposed simulation-based method produced estimates that were highly dependent on the site recruitment strategy (*α_power_*). Under a baseline random sampling strategy (*α_power_* = 0), our method required approximately 320 facilities, an estimate consistent with the standard ICC-adjusted method. This demonstrates that our model is well-calibrated under basic assumptions. However, the key finding is that this site requirement is not fixed. By modeling a strategic, volume-weighted recruitment approach, the estimated number of required facilities was dramatically reduced to approximately 150 with a moderately weighted strategy (*α_power_* = 1) and to just 110 with a strongly weighted strategy (*α_power_* = 2). These results show that a strategic approach allows the target precision to be achieved with substantially fewer sites than predicted by the static analytical methods.

### 3.2 Consistency of findings across scenarios

**Fig. 2:**
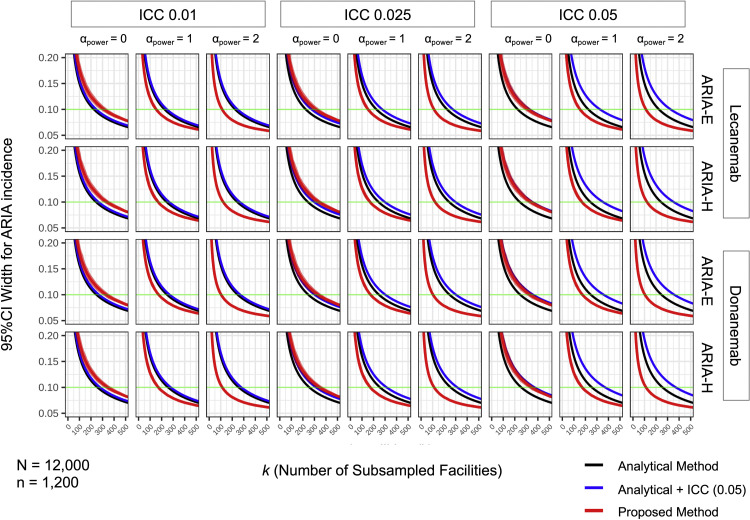
**Comprehensive Comparison of Estimation Methods Across Multiple Scenarios**. A comprehensive comparison of the proposed simulation-based method (red), the conventional analytical method (black), and the intra-class correlation coefficient (ICC)-adjusted analytical method (blue) across a wide range of clinical and methodological scenarios. Each panel displays the 95 % CI width for ARIA incidence as a function of the number of participating facilities. The rows compare results for different drugs (lecanemab, donanemab) and ARIA types (ARIA-E, ARIA-H). The columns show the effect of varying the assumed ICC (= 0.01, 0.025, and 0.05) for the analytical adjustment, and the recruitment strategy (α_power_ ​​= 0, 1, 2). All scenarios are based on a total patient population of *N* = 12,000 distributed across *n* = 1200 facilities with *a_2_* = 0.15, *Adjust p_2_* = 1.0, *ZTNB size* = 0.5, and *ZTNB* μ = 0.5 × *N/n*.

Abbreviations: ARIA, Amyloid-Related Imaging Abnormalities; ICC, intra-class correlation coefficient.

To ensure the robustness of these findings, we examined their consistency across a wide range of clinical and methodological scenarios (Fig. 2). The key patterns observed in the representative scenario remained consistent across all tested conditions. Specifically, we confirmed that: (1) the naive conventional method consistently underestimated site requirements compared to the other approaches; (2) under random sampling, our proposed method provided estimates comparable to the standard ICC-adjusted method; and (3) volume-weighted sampling (*α_power_* > 0) within our framework consistently offered the most efficient path to achieving target precision. This held true when varying the drug (lecanemab or donanemab), the ARIA type (ARIA-E or ARIA-H), and the assumed ICC value (0.01, 0.025, or 0.05).

### 3.3 Factors influencing precision (Linear regression)

A linear mixed-effects models (Table 1) identified significant predictors of both the baseline precision (CI width at intercept) and the rate of precision improvement with increasing facility numbers (the slope).

**Table 1:** Effects on power level and slope: linear mixed‐effects model estimates ($\times 10^{-3}$).

		Non-interaction effects (on power curve level)			Interaction effects with *k* (on power curve slope)		
		Coefficient	Lower 95 %	Upper 95 %	Coefficient	Lower 95 %	Upper 95 %
(Intercept)		1287	1198	1376			
Number of Sites (*k*)		−1.304	−1.37	−1.239			
Being our proposed method		−878	−916	−839	−0.785	−0.822	−0.748
Number of Patients (*N*)	6000	(reference)			(reference)		
	9000	−29.861	−31.735	−27.986	0.03	0.028	0.032
	12,000	−47.496	−49.391	−45.601	0.049	0.047	0.051
	18,000	−66.45	−68.378	−64.521	0.071	0.07	0.073
	24,000	−76.072	−78.026	−74.118	0.085	0.083	0.087
	30,000	−81.277	−83.249	−79.305	0.093	0.091	0.095
Number of Facilities (*n*)	600	(reference)			(reference)		
	900	22.337	20.088	24.586	0.365	0.342	0.388
	1200	40.346	37.994	42.698	0.553	0.519	0.586
	1500	56.002	53.523	58.48	0.668	0.628	0.708
*APOE*-ε4 homozygotes prevalence	0.10	5.659	4.313	7.005	−0.039	−0.041	−0.038
	0.15	(reference)			(reference)		
	0.20	−7.150	−8.563	−5.737	0.016	0.014	0.017
Adjustment in Prevalence of ARIA among ε4 homozygotes	p_2_ × 0.9	−3.163	−4.539	−1.787	0.000	−0.001	0.002
	p_2_ × 1.0	(reference)			(reference)		
	p_2_ × 1.1	1.772	0.404	3.14	0.000	−0.002	0.001
Weights in facility sampling (α_power_)	0	(reference)			(reference)		
	1	−50.772	−52.123	−49.421	0.044	0.043	0.046
	2	−71.832	−73.22	−70.445	0.063	0.061	0.064
ZTNB size	0.5	(reference)			(reference)		
	1.0	16.844	15.721	17.966	−0.015	−0.016	−0.013
ZTNB μ	N/*n* × 0.5	(reference)			(reference)		
	N/*n* × 1.0	−45.742	−47.081	−44.404	0.04	0.038	0.041
	N/*n* × 2.0	−76.238	−77.648	−74.827	0.066	0.065	0.068

Abbreviations: ZTNB, zero-truncated negative binomial; ARIA, Amyloid-Related Imaging Abnormalities.

Factors that increased the number of target subjects available for analysis were associated with better baseline precision (i.e., a narrower CI width). This included a larger total patient population (*N*) or a higher prevalence of APOE**-**ε4 homozygotes (*a_2_*). Specifically, increasing the prevalence of APOE-ε4 homozygotes from 15 % to 20 % was associated with an improvement in baseline precision, narrowing the CI width by 0.007. Conversely, conditions that increased caseload heterogeneity, such as distributing the patient population across a larger number of total facilities (*n*), were associated with worse baseline precision (a wider CI). Interestingly, the baseline ARIA incidence rate (*p_2_*) among homozygotes also influenced precision; because the variance of a proportion is greatest at 0.5, incidence rates closer to this value resulted in wider CIs, while rates further from 0.5 resulted in narrower CIs.

Most notably, the recruitment strategy had a profound impact. Weighted sampling (*α_power_* = 1 or 2) significantly improved baseline precision compared to random sampling (αpower =0). For example, a moderately weighted strategy (*α_power_* = 1) was associated with a 0.051 decrease in CI width compared to random sampling (*α_power_* = 0).

The analysis of the slope (the interaction effects with k) revealed how quickly precision improved as more facilities were added. The rate of improvement was significantly faster for our proposed method compared to the analytical methods (interaction coefficient = −0.000785). This indicates that while our method yields a more conservative (wider) CI at baseline under random sampling, the marginal gain in precision from recruiting each additional facility is greater under our simulation framework. Furthermore, a larger total patient population (*N*) or a higher number of total facilities (*n*) showed effects to flatten the slope, suggesting that the marginal benefit of adding one more facility is diminished when the overall scale of the registry is larger.

## 4 Discussion

This study demonstrates that conventional approaches to estimating required site numbers—such as dividing total patient requirements by the average number of patients per site—may provide inflexible and potentially misleading projections because they cannot account for the impact of real-world recruitment strategies. Our simulation-based method, by modeling different recruitment scenarios, reveals a more nuanced picture. Under a baseline random recruitment strategy (*α_power_* = 0), our simulation produced an estimate (∼320 sites) that was consistent with the standard ICC-adjusted analytical method, validating our framework against established techniques. Both of these estimates were substantially higher than that of the naive conventional method (∼220 sites).

The key advantage of our framework is its ability to model and quantify the benefits of realistic, strategic recruitment efforts. Our results show that when a volume-weighted recruitment strategy is employed, the number of facilities required to achieve a target precision is substantially lower than the estimates provided by both conventional and ICC-adjusted analytical methods. For instance, a strongly weighted approach (*α_power_* = 2) reduced the estimated site requirement to ∼110 facilities, a number far more efficient than the ∼220 to ∼320 facilities suggested by analytical methods.

This finding repositions our method not as a conservative tool, but as a realistic optimization tool. The discrepancy between our α > 0 results and the analytical estimates arises because our simulation correctly captures the efficiency of targeting high-volume centers, a crucial real-world factor that static formulas ignore. When high precision is required, leveraging this strategic approach is critically important.

Existing literature on real-world studies of anti-amyloid therapies is only beginning to address the challenges of multicenter enrollment and safety monitoring (e.g., ALZ-NET [5]; the AD-DMT Registry). To our knowledge, no prior work has provided a framework for optimizing site selection based on caseload heterogeneity, moving beyond the simplistic "total patients ÷ average per center" rule. Our findings highlight that simplistic approaches provide an inflexible estimate that fails to account for the impact of recruitment strategy. Recent early real-world studies of lecanemab provide context [10]: for instance, an initial single-center experience with 71 lecanemab patients reported ARIA-H in 9 patients (12.7 %) and ARIA-E in 7 patients (9.9 %), with a combined 17 % of patients experiencing some ARIA event. Notably, 44 % of *APOE* ε4-homozygotes in that cohort developed ARIA, compared to 17 % of heterozygotes, mirroring the elevated risks seen in trials. This reinforces the need for well-planned registries capable of estimating these risks with adequate precision, a goal our framework helps to achieve more efficiently.

Our proposed site-based simulation analysis has direct implications for ongoing and upcoming Alzheimer’s real-world registries—such as the Japanese AD‐DMT registry or ALZ-NET in the U.S. They could use this method to guide how many sites to recruit and which sites to prioritize. Our weighted recruitment scenarios showed a dramatic reduction in required centers when preferentially selecting top-enrolling sites. On the other hand, real-world registries also have goals of inclusiveness and representativeness. Our method provides a tool to balance these priorities: study planners can experiment with different *α_power_*-weighting schemes to evaluate trade-offs between broad geographic coverage and statistical efficiency.

This study has several limitations. First, ARIA frequencies were based primarily on clinical trials involving predominantly White populations; rates in other populations (e.g., Asians including Japanese) might differ (or be lower), potentially requiring more centers than estimated here. Second, the patient populations in real-world settings may differ from clinical trial populations, affecting the generalizability of the ARIA frequencies and *APOE* distributions employed in this study. Third, the ZTNB distribution parameters (size, μ) used to model patient caseload variability were assumed, which will require validation against actual registry data. Fourth, the simulation assumed complete enrollment of all eligible patients at participating centers, which may not hold true in practice, potentially leading to an underestimation of the estimated number of required centers. Fifth, our model assumes homogeneity in patient risk profiles across sites, whereas factors like facility size could correlate with case severity. And lastly, the proposed method requires assumptions about distribution parameters (ZTNB size, μ) which are often unknown a priori when starting a novel registry project, introducing potentially more uncertainties compared to the conventional method, although sensitivity analyses (Table 1) suggested these parameters had relatively smaller impacts.

While registries like ALZ-NET and the AD-DMT Registry have focused on data collection and safety outcomes but have not published detailed power analyses addressing how many sites are needed. By comparing our method’s projections to conventional estimates, we demonstrate how strategic site selection and weighting can improve the efficiency of a study. Future work should also empirically calibrate ZTNB parameters using existing registry data and extend the framework to incorporate patient‐level covariates (e.g., age, comorbidities) and adaptive center‐recruitment algorithms. Integrating dynamic enrollment monitoring could enable mid‐study adjustments to recruitment targets, further safeguarding against imprecise estimates in evolving real‐world settings.

In conclusion, our study provides a novel, site-centered perspective on sample size planning for multicenter real-world studies of AD treatments. Its primary contribution is to provide a more realistic and flexible planning tool. By demonstrating that strategic recruitment can substantially reduce site requirements compared to estimates from conventional methods, this approach offers more reliable guidance on “how many sites are enough”–a question of growing importance as anti-amyloid therapies are rolled out in clinical practice worldwide. With further refinement and the incorporation of emerging real-world data, this framework can be an integral part of planning safe and effective post-marketing surveillance for AD and beyond.

## CRediT authorship contribution statement

**Kenichiro Sato:** Writing – original draft, Formal analysis, Data curation, Conceptualization. **Yoshiki Niimi:** Writing – review & editing, Investigation, Funding acquisition. **Ryoko Ihara:** Writing – review & editing, Conceptualization. **Atsushi Iwata:** Writing – review & editing, Conceptualization. **Takeshi Iwatsubo:** Writing – review & editing, Supervision, Conceptualization.

## Declaration of competing interest

The authors declare that they have no known competing financial interests or personal relationships that could have appeared to influence the work reported in this paper.

The authors declare the following financial interests/personal relationships which may be considered as potential competing interests:

Takeshi Iwatsubo reports financial support was provided by Effissimo Capital Management Pte Ltd. Takeshi Iwatsubo reports financial support was provided by Japan Agency for Medical Research and Development. Kenichiro Sato reports financial support was provided by Japan Society for the Promotion of Science. If there are other authors, they declare that they have no known competing financial interests or personal relationships that could have appeared to influence the work reported in this paper.

## Appendix Supplementary materials

Image, application 1
