# Supplementary material for: How many sites are enough? a novel, site-based power analysis method for real-world registry studies of anti-amyloid monoclonal antibodies
Source: JAR Life. 2025 Jul 22;14:100020. doi: 10.1016/j.jarlif.2025.100020 (PMC12304769; doi:10.1016/j.jarlif.2025.100020)
Supplement: Supplementary file 1 [file mmc1.docx]

Supplementary Materials

**Table S1. Applied ARIA Prevalence Parameters (p_2_, p_1_, p_0_)**

| Drug | ARIA type | *APOE*-ε4  genotype | Prevalence of ARIA [0-1] | Odds Ratio of  ARIA development in ε4-homozygotes (vs. negatives) |
| --- | --- | --- | --- | --- |
| Lecanemab | ARIA-E | Homozygote | p_2_ = 0.326 | 8.473 |
|  |  | Heterozygote | p_1_ = 0.109 |  |
|  |  | Negative | p_0_ = 0.054 |  |
|  | ARIA-H | Homozygote | p_2_ = 0.390 | 4.733 |
|  |  | Heterozygote | p_1_ = 0.140 |  |
|  |  | Negative | p_0_ = 0.119 |  |
| Donanemab | ARIA-E | Homozygote | p_2_ = 0.405 | 3.655 |
|  |  | Heterozygote | p_1_ = 0.228 |  |
|  |  | Negative | p_0_ = 0.157 |  |
|  | ARIA-H | Homozygote | p_2_ = 0.503 | 4.371 |
|  |  | Heterozygote | p_1_ = 0.323 |  |
|  |  | Negative | p_0_ = 0.188 |  |

Abbreviations: ARIA, Amyloid-Related Imaging Abnormalities.
